# Interstitial nephritis in melanoma patients secondary to PD-1 checkpoint inhibitor

**DOI:** 10.1186/s40425-016-0205-2

**Published:** 2017-01-17

**Authors:** Julia Escandon, Stephanie Peacock, Asaad Trabolsi, David B. Thomas, Ayman Layka, Jose Lutzky

**Affiliations:** 1 Mount Sinai Medical Center, Miami Beach, FL USA; 2 Sylvester Comprehensive Cancer Center, University of Miami, Miami, FL USA; 3 Department of Pathology, University of Miami, Miami, FL USA; 4 Melanoma Program, Division of Hematology/Oncology, Mount Sinai Comprehensive Cancer Center, 4306 Alton Road, Miami Beach, FL 33140 USA

**Keywords:** Immune checkpoint inhibitor, Programed death 1 receptor (PD-1), PD-1 ligand (PD-L1), Interstitial nephritis, Pembrolizumab, Nivolumab

## Abstract

**Background:**

Immune checkpoint inhibitors have become the first line therapy in melanoma treatment and their use is extending to other malignancies. However, we are still learning about immune side effects produced by these drugs and their severity especially in patients with history of inflammatory diseases.

**Case presentation:**

We present two cases of metastatic melanoma treated with nivolumab and pembrolizumab (anti PD-1). Both patients developed acute interstitial nephritis during immune checkpoint therapy. We emphasize the causal association between immune checkpoint inhibitors and the nephritis. The timing of drug administration and appearance of nephritis is suggestive of a causal relation between the checkpoint inhibitor therapy and this adverse event.

**Conclusions:**

Although uncommon, some side effects from checkpoint inhibitors can be severe and may need to be addressed with immunosuppression. Given the increasing frequency of immunotherapy use, awareness should be raised in regards to immune side effects and their appropriate management.

## Background

Programed death 1 receptor (PD-1), transmits inhibitory signals to immune cells leading to decreased proliferation and apoptosis. Cancer cells express PD-L1, the ligand of PD-1, allowing the tumor to escape attack by effector T cells. Immune checkpoint inhibitors encompass blocking antibodies to the PD-1/ PD-L1 and CTLA-4 checkpoint molecules. These drugs block the PD-1/PD-L1 interaction enhancing the cellular response against the tumor.

Pembrolizumab, an anti-PD-1 antibody, has shown superior progression-free survival (PFS) versus chemotherapy at 6 months: 34–38% versus 16% PFS, respectively [1]. Nivolumab, another anti-PD-1 antibody, approved by the FDA in 2014 to treat metastatic melanoma. A trial comparing nivolumab to dacarbazine in previously untreated patients showed a 12-month survival rate of 73% versus 42% respectively [2]. Another study compared nivolumab to dacarbazine or carboplatin/paclitaxel in patients who progressed on ipilimumab plus or minus BRAF-inhibitor; results showed response rates of 31.7% versus 10.6% respectively [3].

Nivolumab, alone or in combination with ipilimumab, and pembrolizumab as a single agent are indicated to treat metastatic melanoma [4]. Anti-PD-1 antibodies are now standard treatment for metastatic or unresectable melanoma regardless of BRAF V600 mutation status and are also indicated in non-small cell lung cancer and renal cell carcinoma. Drug-related adverse events are mostly Grades 1 and 2; Grade 3 or 4 toxicity occurred in only 11.7% of patients treated with nivolumab and 17.6% of those treated with dacarbazine [2].

For nivolumab, serious side effects include colitis, hepatitis, hypophysitis, adrenal insufficiency, type 1 diabetes mellitus, nephritis, extensive rash and encephalitis [5]. Side effects of pembrolizumab include pneumonitis, colitis, endocrinopathies, nephritis and transaminases and bilirubin elevation [6].

Anti PD-1 and anti CTLA-4 combined therapy clinical trials also noted the occurrence of renal adverse events. For instance, the CheckMate-069 trial combined Ipilimumab 3 mg/kg (anti CTLA-4) with Nivolumab 1 mg/kg and reported a proportion of 1:1000 cases of renal failure of grade 1-2 while Ipilimumab 3 mg/kg or Nivolumab 3 mg/kg alone reported 0:1000 and 2:1000 cases of renal failure respectively [7].

Here, we present two patients with advanced melanoma that developed interstitial nephritis during treatment with anti-PD1 antibodies.

## Cases presentation

Patient 1: A 64-year-old Caucasian male with a history of metastatic melanoma and prostate cancer in remission. Melanoma was diagnosed on the left forearm in December 2008; it was stage IB, Breslow depth 1.2 mm, non-ulcerated, 2 mitoses/mm^2^. He was treated with a wide local excision and sentinel lymph node biopsy was negative for metastasis. He was followed until February 2010 and subsequently lost to follow up. In June 2015, he was referred back by a thoracic surgeon who biopsied two tracheal pigmented metastatic lesions. Evaluation with PET/CT demonstrated metastasis involving subcutaneous tissue, lymph nodes, right adrenal and the trachea. MRI showed no brain involvement. In September 2015, pembrolizumab was started at 2 mg/kg every 3 weeks; by December 2015, he had completed 5 cycles with resolution of all lesions except for the right adrenal as evidenced by PET/CT. Complete biochemical profile was obtained before each cycle.

**Fig. 1:**
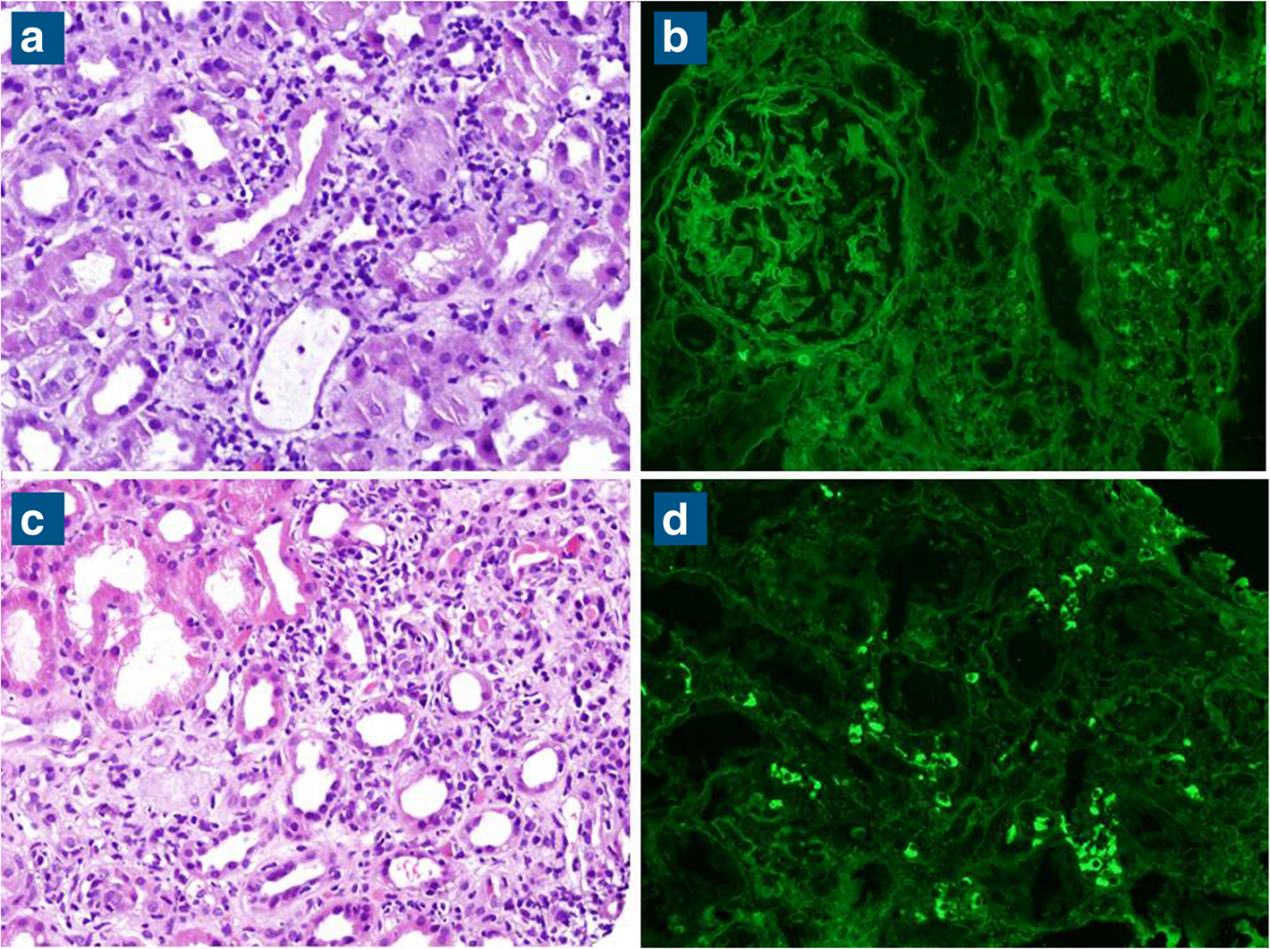
**a**- **b** patient 1. **c**-**d** patient 2. **a** and **c** H&E 20x. Tubulointerstitial inflammation with eosinophils and acute tubular epithelial cell injury. **b** and **c** DIF 20x. IgG reactive interstitial plasma cells with tubulointerstitial inflammation

**Fig. 2:**
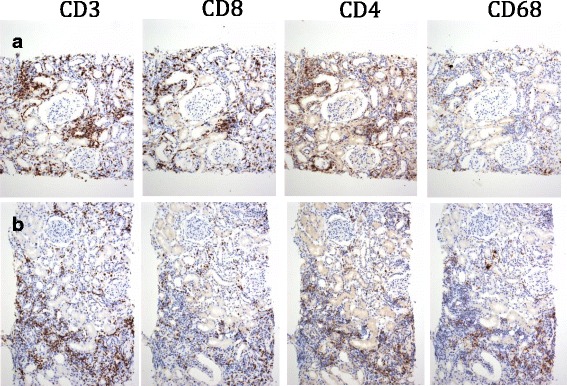
Immunohistochemistry of renal biopsies from patients 2 (*top* panel: **a**) and 1 (*bottom* panel: **b**) reveals an inflammatory infiltrate composed of CD4 and CD8 T-cells and macrophages.

At the time of his sixth scheduled cycle he presented with a serum creatinine of 4.3 mg/dl (baseline Cr: 0.9–1 mg/dl) and was admitted to the hospital. He had no history of kidney disease, hypertension, diabetes, gout, kidney stones or frequent UTIs. His outpatient medications were: fentanyl patch, hydromorphone, hydrocodone-acetaminophen, lorazepam, omeprazole, temazepam and montelukast. He also reported occasional use of NSAIDs. He denied recent upper respiratory symptoms, hematuria, dysuria, foamy urine, abdominal pain, nausea, vomiting, diarrhea, GI bleed, mouth ulcers or inflammatory joint disease. Inflammatory markers and rheumatologic workup were performed (Table 1). He underwent a kidney biopsy and was started on IV methylprednisolone 1 g/day for three days followed by oral prednisone 60 mg/day. Renal biopsy (Fig. 1) demonstrated diffuse active tubulointerstitial nephritis with severe acute tubular cell injury. Light microscopy showed mononuclear interstitial inflammation with plasma cells and eosinophils. Immunohistochemistry revealed an inflammatory infiltrate composed of CD4 and CD8 T-cells and macrophages (Fig. 2).

**Table 1:** Diagnostic tests in acute renal insufficiency after checkpoint inhibitors

	Case 1 (male)	Case 2 (female)	Reference range
Serology			
ANA titer	<40	<40	Negative
CRP	**97**	**37.9**	0–3.0
ESR	123	50	0–30
ASO	47	32	<200
Anti dsDNA by EIA	7	19	Negative
ANCA SCR W/MPO/PR3 W/REFLEX	Negative	Negative	Negative
ANA screen - serum	Negative	Positive	Negative
Kappa Light Chains Free light chains	65.26	30.59	3.3–19.4
Lambda light chain, free	29.59	19.03	5.7–26.3
Kappa/ Lambda free	2.45	0.26–1.65	1.61
Hepatitis profile – Acute panel	Negative	Negative	Negative
HAV IgM, HCV Ab, ABB Core IGM, HBV Surface AG			
RPR	Non-reactive	Non-reactive	Non-reactive
SM/RNP Antibodies	<0.1	<0.1	<0.1
SM Antibodies	<0.1	<0.1	<0.1
CPK	38	35	26–192
C3	149	150	90–180
C4	39.8	46.5	10.0–40.0
Uric Acid	6.5	9.5	2.6–6
Vitamin D 25	13.6	32.6	30–100
PTH intact	105.6	79.2	11.1–79.5
Calcium	8.9	8.9	8.5–10.1
Protein electrophoresis with immunofixation			
Protein total	7.8	6.8	6.4–8.2 g/dl
Albumin	3.72	4.53	3.57–5.42 g/gl
Alpha 1	**0.66**	**0.46**	0.19–0.4 g/dl
Alpha 2	**1.44**	**1.11**	0.45–0.97 g/dl
Beta 1	0.47	0.44	0.3–0.59 g/dl
Beta 2	0.56	0.46	0.21–0.53 g/dl
Gamma	0.94	0.79	0.71–1.54 g/dl
Urinalysis			
Color	Straw	Yellow	Yellow
Turbidity	Slightly cloudy	Clear	Clear
Glucose	Negative	Negative	Negative
Bilirubin	Negative	Negative	Negative
Ketones	Negative	Negative	Negative
pH	6.0	6.5	4.5–8
Protein	Negative	Negative	Negative
Specific gravity	1.005	1.01	Up to 1.035
Blood	Small	Negative	Negative
Urobilinogen	Negative	0.2	Negative
Nitrites	Negative	Negative	Negative
Leukocyte esterase	Large	Negative	Negative
WBC, UA	38	Negative	Negative
RBC, UA	3	Negative	Negative
UBAC	+1	Negative	Negative
Budding Yeast	Present	Negative	Negative
White blood cell clump	Present	Negative	Negative

Data in boldface represent abnormal results

Here was no hypercellularity, necrosis, crescents or interstitial fibrosis. Moderate arteriosclerosis. Immunofluorescence showed only trace C3 staining in mesangium and electron microscopy revealed mild segmental podocyte foot process effacement 30%. No electron dense deposits or endothelial tubuloreticular inclusions were seen. Creatinine was 2.45 mg/dl at discharge and steroids were tapered over 6 weeks. Upon discontinuation of steroids the renal function returned to baseline. He was not restarted on pembrolizumab. However, ipilimumab was started in July 2016 due to disease progression.

Patient 2: A 78-years-old Hispanic female with history of acral melanoma, hypertension, psoriasis and arthritis was admitted with worsening renal function after three cycles of nivolumab. In September 2013, acral melanoma was diagnosed in the left fourth toe; Breslow depth was 15 mm, non-ulcerated. PET/CT scan suggested metastases to left groin lymph nodes. In November 2013, she underwent amputation of the second and third left toes with negative margins; sentinel node biopsy was positive for 3/3 nodes. In December 2013, completion lymphadenectomy resulted in 1/4 positive nodes for a total of 4/7 positive lymph nodes. She declined adjuvant therapy and underwent active surveillance. In May 2015, the patient presented with a new subcutaneous nodule on the anterior left leg (Fig. 2), which was confirmed as metastatic melanoma. PET/CT scan revealed multiple in-transit lesions along the left lower extremity. In July 2015, nivolumab was started at 3 mg/kg. Her baseline serum creatinine was 0.75 mg/dl. After three cycles, nivolumab was discontinued because of Grade 3 cutaneous toxicity. Subsequently, a steady increase in the serum creatinine level was noted. The level in October was 0.92, reaching 3.14 by December 2015 when she was admitted to the hospital. Her medications at the time of admission included: clonidine, atorvastatin, lorazepam, ferrous sulfate, nifedipine, omeprazole, hydrocodone-acetaminophen, butalbital-acetaminophen-caffeine; she denied taking NSAIDs or nephrotoxic medications and her hypertension was controlled. She had no history of diabetes or family kidney disease. Complete workup for renal and rheumatologic disease was done similarly to the previous case (Table 1). She underwent renal biopsy and was started on IV methylprednisolone 1 g/day for 3 days followed by oral prednisone 60 mg daily. Renal biopsy revealed diffuse active on chronic tubulointerstitial nephritis with acute tubular cell injury. Light microscopy showed a mononuclear interstitial inflammation with lymphocytes, plasma cells and eosinophils and no hypercellularity, necrosis or crescents. There was mild interstitial fibrosis with mild tubular atrophy and mild arteriosclerosis. Immunofluorescence revealed no glomerular, tubular or vascular wall immune staining (Fig. 1). Immunohistochemistry revealed an inflammatory infiltrate composed of CD4 and CD8 T-cells and macrophages (Fig. 2). Electron microscopy demonstrated minimal podocyte foot process effacement and no electron dense deposits. Discharge renal function was improved (Cr: 1.53 mg/dl) and steroids were tapered down and stopped after 6 weeks when serum creatinine had normalized (Cr: 1.0 mg/dl). She was not restarted on nivolumab and by June 2016 she had completed three cycles of temozolomide.

## Discussion

As the use of immunotherapy in the treatment of melanoma and other malignancies increases, infrequent but serious adverse events will become more prevalent. In these two cases treated with anti-PD-1 antibodies, one patient received pembrolizumab and the other received nivolumab. Manufacturer information warns of renal function compromise in 5% of patients treated with nivolumab (40/787 patients pooled from clinical trials), of which 0.8% (6/787 cases) presented with Grade 2 and Grade 3 toxicity. All cases had complete resolution of the adverse event. Three of them permanently stopped nivolumab [5]. In the case of pembrolizumab, manufacturer data reported nephritis in 0.4% of patients (7/1567 patients pooled from clinical trials), and these included Grades 2, 3 and 4 nephritis. Two patients (0.1%) permanently discontinued pembrolizumab and four out of seven patients had resolution of nephritis [6].

In our case report, both patients were routinely followed in the clinic with laboratory tests prior administering immunotherapy and the rise in creatinine prompted further workup for kidney pathology. The differential diagnosis for an insidious rise in creatinine in patients with no previous history of kidney disease includes prerenal (hypovolemia, rhabdomyolysis), renal (rheumatologic entities, post infectious glomerulopathies, nephrotoxic medication) and postrenal (urinary system obstruction) etiologies, for which complete work up was negative. In the first patient, the timing of the rise in creatinine levels and the introduction of pembrolizumab in the absence of any other precipitating factor (other new drugs, infection) with lack of constitutional symptoms, pointed to immunotherapy being the most likely cause. In the second patient, the largest increase in creatinine occurred after nivolumab had been discontinued; nevertheless, the median time for occurrence of renal adverse events with nivolumab has been reported to be 15.1 weeks and 85% of these cases occurred within 16 weeks of treatment [8].

Regarding concomitant medications, our first patient had been using PPI/NSAIDs for many years. However, AKI only occurred at the time of introducing immunotherapy. The second patient had been using PPI for many years before and after the introduction of nivolumab. It is unlikely that PPI is the cause of autoimmune nephritis unless immunotherapy played a role by adjusting immune response to PPIs.

Results from both renal biopsies were quite similar. Immune complex deposition diseases were ruled out by histology; absence of necrosis and granuloma formation ruled out ischemia and mycobacterial infection respectively and no infectious collections were seen. Absence of crescents allayed the concern for lupus nephritis, post infectious or membranoproliferative glomerulonephritis, IgA nephropathy, etc. Remarkably, the biopsies presented interstitial infiltration with lymphocytes, plasma cells and eosinophils which are the most likely culprits of the nephritis.

The reported CD4+/CD8+ T lymphocyte infiltrate in the setting of treatment with immunotherapy raises the hypothesis of whether the effector T cells were involved in provoking kidney injury. PD1 checkpoint inhibition stimulates the T cell effector response, consistent with the infiltration of immune cells in the renal parenchyma [9].

Our second patient presented with low titer positive ANA. This is perhaps reflective of autoimmunity in the setting of immunotherapy or simply an incidental finding, not uncommon in the elderly.

## Conclusion

Checkpoint inhibitor immunotherapy is highly effective in treating melanoma and other malignancies. Serious immune adverse events are uncommon, but their numbers will increase as usage of these drugs becomes more prevalent. While renal compromise has been previously documented, it is warranted to recognize that interstitial nephritis may present with negative serological, urine and urine microscopy tests and that it may be only be fully identified with a kidney biopsy as in the cases we report here. Close monitoring and a high degree of clinical suspicion is recommended in patients treated with immune checkpoint inhibitors and discontinuation of the drug is suggested depending on the severity of the adverse event. For most grade 1 and 2 toxicities, drug can be safely but carefully restarted. Patient re-treatment with immunotherapy after grade 3 toxicity requires close evaluation of the risks. Patients with grade 4 toxicity should not be re-challenged.

## Abbreviations

PD-1: Programed death 1 receptor
PD-L1: Programmed death 1 receptor ligand.
